# Mental Health in the Time of COVID-19 Pandemic: A Worldwide Perspective

**DOI:** 10.3390/ijerph19010161

**Published:** 2021-12-24

**Authors:** Gaia Sampogna, Maurizio Pompili, Andrea Fiorillo

**Affiliations:** 1 Department of Psychiatry, University of Campania “L. Vanvitelli”, 80138 Naples, Italy; gaia.sampogna@gmail.com; 2 Suicide Prevention Centre, Department of Neurosciences, Mental Health and Sensory Organs, Faculty of Medicine and Psychology, Sant’Andrea Hospital, Sapienza University of Rome, 00185 Rome, Italy; maurizio.pompili@uniroma1.it

Major infectious disease outbreaks, such as the novel coronavirus (COVID-19) pandemic, create significant distress for the general population, and pose a heavy burden on the healthcare systems called to care for affected individuals and contain the spread of the disease [1,2]. 

The current pandemic does not represent the first pandemic witnessed in the history of mankind, rather it represents the first one in the era of globalization, digitalization, and commodification. These are just some of the main features of modern society that have contributed to the spread of the pandemic and, at the same time, to its profound impact on the mental health of the general population [3,4].

The COVID-19 pandemic is more than a health crisis; it is a period characterized by deep fears, worries, and uncertainty, which heavily influence public behaviors. Several concerns have been reported from the general population, ranging from the threat to personal and family safety, to the difficulties in receiving a prompt diagnosis of the infection, to the use of containment measures that limit personal mobility, to the lack of effective treatments to be used [5,6]. The availability of vaccines has improved the situation significantly, but has also been associated with a variety of partially unexpected complications, as well as generating new inequities [7].

Taking into account these premises, it has been clearly and repeatedly stated that the COVID-19 pandemic has had a profound negative impact on the physical and mental health of the general population [8,9,10]. The pandemic can be considered as a new type of traumatic stressor, being an unexpected event, affecting the whole population worldwide and causing severe disruption to daily routines [11,12]. 

During this pandemic, traumatic stress reactions, including intrusive re-experiencing and heightened arousal, are frequent [13], and may be due to the direct threats to important life resources of the general population, such as safety, health, income [14,15], work, housing, and social support [16,17]. Furthermore, the traumatic stress reactions to the COVID-19 pandemic have been worsened since the beginning by indirect exposure, e.g., via mass-media coverage and the infodemic phenomenon [18]; by the psychosocial consequences, in terms of unemployment, isolation [19], suicide/suicidal behaviors [20,21,22]; by the lack of clear and consistent guidelines about how to avoid and manage the infection [23]. 

The consequences of the pandemic are slightly different according to the target population considered. In particular, a high prevalence of mental exhaustion, burn-out syndrome, and insomnia/sleep disorders has been found in healthcare workers [24,25,26]. In disabled people and in those with pre-existing mental health problems, an increased risk of interruption to long-term treatments has been observed, associated with relapses or symptoms worsening, as well as with a higher risk of being infected with COVID-19 [27,28,29,30,31]. 

The specific risk factors identified for the development of these mental health disturbances include female gender, having had previous psychiatric or physical disorders, loneliness, time spent on the internet, and unemployment [32,33].

In the general population, high levels of distress [34] and post-traumatic reactions [35,36,37] have been found. Moreover, a specific type of health anxiety disorder related to COVID-19 has been described [38], which is associated with a higher risk of accessing mental health services in the long term. 

In young people and adolescents, social isolation with suicidal ideation [39,40,41,42], depressive and anxiety symptoms, as well as sleep disorders [43,44,45,46,47] have been found to be more frequent. 

All these special populations should be carefully taken into consideration, and their mental health needs should be evaluated and addressed through ad hoc interventions. In particular, people living with disabilities, or those affected by chronic physical and mental health disorders [48], require dedicated interventions, which are completely different from those needed, for instance, by pregnant women [49,50,51,52,53], the elderly [54,55], or young people [56,57,58,59]. 

These different populations are exposed to the same traumatic event (i.e., the pandemic), but its perception is highly variable, since it can be mediated and moderated by individual psychological and social factors, such as coping strategies and resilience styles [60,61,62,63,64]. For this reason, it is essential to develop ad hoc strategies targeting resilience, coping styles, and problem solving, in order to mitigate the negative effects of the pandemic.

The global crisis due to the COVID-19 pandemic started almost two years ago, and the spread of new variants of the virus has caused an increase in the infection rates, with the need for new lockdowns and adoption of further severe containment measures. The detrimental effects of the pandemic remain multifaceted, ranging from the global economic downturn to the extreme burden posed on healthcare systems worldwide, and to the risks related to the forced isolation due to lockdown measures [65]. These measures have been necessary in order to contain and limit the spread of the pandemic during its initial phase, but it is now necessary to evaluate their impact on mental health more effectively, and to think about how to “re-start”. 

In this Special Issue, entitled “Mental health in the time of COVID-19”, several studies, carried out in different parts of the world, such as China [66], Canada [67], the UK [68], Greece [69], Poland [70], Norway [71], India [72], Mexico [73], Malaysia [74], Kenya [75], Saudi Arabia [76], and Switzerland [77], describe the impact of the pandemic on different dimensions of people’s mental health. 

We hope that the data collected in this issue will be useful to disentangle the complex relationship between this new form of trauma and its long-term consequences on mental health. Moreover, these data could be useful to inform policy makers and international scientific societies [78,79,80,81,82,83], guiding them in the development of supportive strategies and interventions to address the long tail of consequences on mental health, and to improve the management of future events with a comparable worldwide impact.

## Data Availability

Not applicable.
